# Hand hygiene and biomedical waste management among medical students: a quasi-experimental study evaluating two training methods

**DOI:** 10.1186/s12909-023-04617-2

**Published:** 2023-09-04

**Authors:** Imen Mlouki, Souha Ben Ayed, Faouzia Chebbi, Nejla Rezg, Aida Khouildi, Amel Haj Sassi, Sana El Mhamdi

**Affiliations:** 1 Faculty of Medicine of Monastir, University of Monastir, N13, Habib 9, Omrane city, Monastir, 5000 Tunisia; 2 Department of Preventive and Community Medicine, Taher Sfar University Hospital, Mahdia, 5100 Tunisia; 3 Research laboratory “Epidemiology Applied to Maternal and Child Health” 12SP17, Monastir, Tunisia

**Keywords:** Hand Hygiene, Medical waste disposal, Medical students, Education, Comparative study, Tunisia

## Abstract

**Background:**

Several studies revealed that medical students have low performance levels of hand hygiene (HH) and biomedical waste management (BMWM). However, there have been limited interventions directed at young students targeting HH and BMWM enhancement. Given these data, we aimed at assessing HH and BMWM among medical students after two training methods.

**Methods:**

We performed a quasi-experimental study from September 2021 to May 2022, which included fifth-year medical students enrolled in the faculty of Medicine of Monastir (Tunisia). We relied on a conventional training based on presentations and simulations guided by the teacher and a student-centred training method based on courses and simulated exercises prepared by students. We used the WHO HH Knowledge Questionnaire and the “BMWM audit” validated by The Nosocomial Infection Control Committee in France.

**Results:**

A total of 203 medical students were included (105 in the control group and 98 in the experimental group) with a mean age of 23 ± 0.7 years. Regarding HH, we found a statistically significant increase in post-test scores for both training methods. A higher post-test mean score was noted for student-centred method (14.1 ± 1.9 vs. 13.9 ± 2.3). The overall improvement in good HH knowledge rates was greater after student-centred method compared to conventional training (40.5% vs. 25%). Concerning infectious waste, mean scores were higher after student-centred learning in all hazardous waste management steps (25 ± 3.3 vs. 23.6 ± 5.5).

**Results:**

Coupling student-centred teaching and continuous supervision could improve HH and BMWM knowledge and practices among medical students.

## Introduction

Hand hygiene (HH) and biomedical waste management (BMWM) are the two major effective practices to prevent a large proportion of hospital acquired infections [1]. During the current COVID-19 pandemic, it has been proved that these safety measures are crucial to control the crisis and to save millions of lives [2–4]. However, HH as well as BMWM practices remain unsatisfactory in many healthcare settings mainly in developing countries [5–7]. Specifically, several studies revealed that medical students have low performance levels regarding HH [4] and BMWM (8,9). Undergraduate medical students participate in healthcare delivery during their clinical posting, yet their defective practices of BMWM and HH may lead to several harmful outcomes [1]. Therefore, appropriate training programs should be designed in order to improve their deportments [8]. As far as we know, there have been limited studies describing interventions directed at young students targeting HH and BMWM enhancement in the Middle East and North African region [9].

Capacity building by training is recommended as a core component for an effective infection prevention and control program by the World Health Organization [10]. Medical academic institutions are the most favourable learning environment to promote good habits [11]. Indeed, appropriate learning methods allow young students developing skills in order to challenge poor practice and to request better resources when they join health care facilities in future [9, 12].

Currently, the Students centred learning (SCL) has become a worthwhile active training method during which students take responsibility for their learning as they develop critical thinking skills [13]. Based on the results of a 2018-survey including 976 college students in China [14], the SCL method improved both cognitive and practical abilities. A recent study conducted in the United States about a peer-derived medical ethics curriculum, revealed that student involvement in curricular development is beneficial [15].Nevertheless, few papers have been published on SCL in medical education.

Given these data, we aimed at assessing hand hygiene as well as biomedical waste management among all fifth-year medical students enrolled in the Faculty of Medicine of Monastir (Tunisia) before and after conventional training and/or student centred learning.

## Methods

### Study design, setting and sampling

Since the academic year 2019–2020, trainings about hospital hygiene and safety have been introduced in the medical curriculum to fifth-year-student in the Faculty of Medicine of Monastir (Tunisia). A 2-hour workshop about HH and BMWM for groups of 10 students per week, were conducted at the Preventive and Community Medicine Department.

We performed a quasi-experimental study during the academic year 2021–2022 among all fifth-year students. Randomization was impossible since the list of student groups was pre-established by the administration. The two training methods were applied alternatively each week.

Students in control groups received a conventional training in which the teacher started with highlighting the objectives of the training followed by interactive sessions using scenario-based learning, lectures and open discussions. The student centred learning (SCL) consisted in a method based on courses and classroom-based role plays prepared and presented entirely by students. Knowing that students were contacted in advance and informed about tasks distribution, they were asked to animate workshops using different tools like projected slides, roles plays and informative videos. Regarding the distribution of topics, each group was randomly divided into two sub-groups composed of five students. One to animate the HH theme and the other to tackle the BMWM. The teacher guided the students during their preparation phase.

At the end of the session, the teacher made updates to correct and complete what has been presented by the students making sure that both groups acquired equal skills training.

### Data collection and study instrument

HH and BMWM knowledge were evaluated using the same instrument before and after each training program. The measurement tool consisted of two parts. One has its objective the HH knowledge evaluation and the other the BMWM assessment. Information about age, gender and previous training in HH or BMWM were also recorded.

#### Hand hygiene knowledge

Questions were adopted from the WHO HH Knowledge Questionnaire for health care workers [16]. This measurement tool consists of 19 items with multiple choice and “yes” or “no” questions. For each correct answer, one point was given. The maximum obtainable score for knowledge was 19 marks. The scores were expressed in percentage. In fact, a total score of > 75% was considered as good, 50–75% as moderate and < 50% as poor HH knowledge [16].

#### Biomedical waste management knowledge

We used the “BMWM audit” validated by The Nosocomial Infection Control Committee in France [17] evaluating the most frequently generated BMW. The instrument was a 22-item structured questionnaire with 14 items about clinical/infectious waste (contaminated by blood, urine or other hazardous body fluids) and 8 items about general/non-infectious waste. Thus, radioactive, chemical and pharmaceutical wastes were not included.

Each item is an example of biomedical waste to be conditioned using specific colour coded bins “Source separation step” and to be transported for treatment “Destination step”. For the source separation step, the answer was considered correct (one mark for each item) when the student chose the correct packaging. Likewise, one mark was given when the waste was directed to the appropriate destination. For each step, maximum obtainable scores for infectious waste and general waste were 14 and 8 marks respectively.

Students were considered to have a good level of BMWM knowledge if the percent score was 50% or more and a low level of knowledge if less than 50%.

### Statistical analyses

Data entry and analysis were conducted using SPSS; Version 23.0. Qualitative variables were represented by effectives and percentages. To compare percentages before and after training, we used the Chi 2 test (χ2) or Fisher’s exact test. After normal distribution testing, continuous variables were expressed as the mean plus or minus the standard deviation (mean ± standard deviation). Independent sample t-tests were performed to compare the mean scores and change scores of knowledge between the pretest and the post test for each training method at the significant level of 0.05.

## Results

### Characteristics of the study participants

During the academic year 2021–2022, a total of 390 final-year medical students participated in the training program. Of those, 203 returned the questionnaires (105 in the control group and 98 in the experimental group) with an overall response rate of 52%. The majority of them were females (72.4%) with a mean age of 23 ± 0.7 years.

### Hand Hygiene knowledge among medical students before and after conventional training and student-centred methods

Out of the 203 participants, 21.7% had undergone prior training in the last three years for HH. About 90% of them routinely used an alcohol-based handrub for hand hygiene.

Details about scores during different phases of workshops according to HH training method are presented in Table 1.

There was a statistically significant increase in post-test scores compared to pre-test scores for both training methods. The student-centered method resulted in a higher post-test mean score (14.1 ± 1.9) compared to conventional training (13.9 ± 2.3) (Table 1).

**Table 1:** Hand hygiene knowledge scores before and after the two training methods

Hand hygiene training method	Conventional training (n = 105)			Student-centred training (n = 98)		
	Pre-test HH	Post-test HH	P-value	Pre-test HH	Post-test HH	P-value
Mean score ± Standard Deviation (Maximum score = 19)	11.5 ± 2.2	**13.9 ± 2.3**	**< 0.001**	10.8 ± 2.1	**14.1 ± 1.9**	**< 0.001**

After conventional training, no significant difference in good HH knowledge levels was found according to gender (24.1% for males vs. 23.6% for females; p = 0.4). Similarly, 25.9% of males compared to 26.3% of females (p = 0.8) had good HH knowledge after SCL method.

**Fig. 1:**
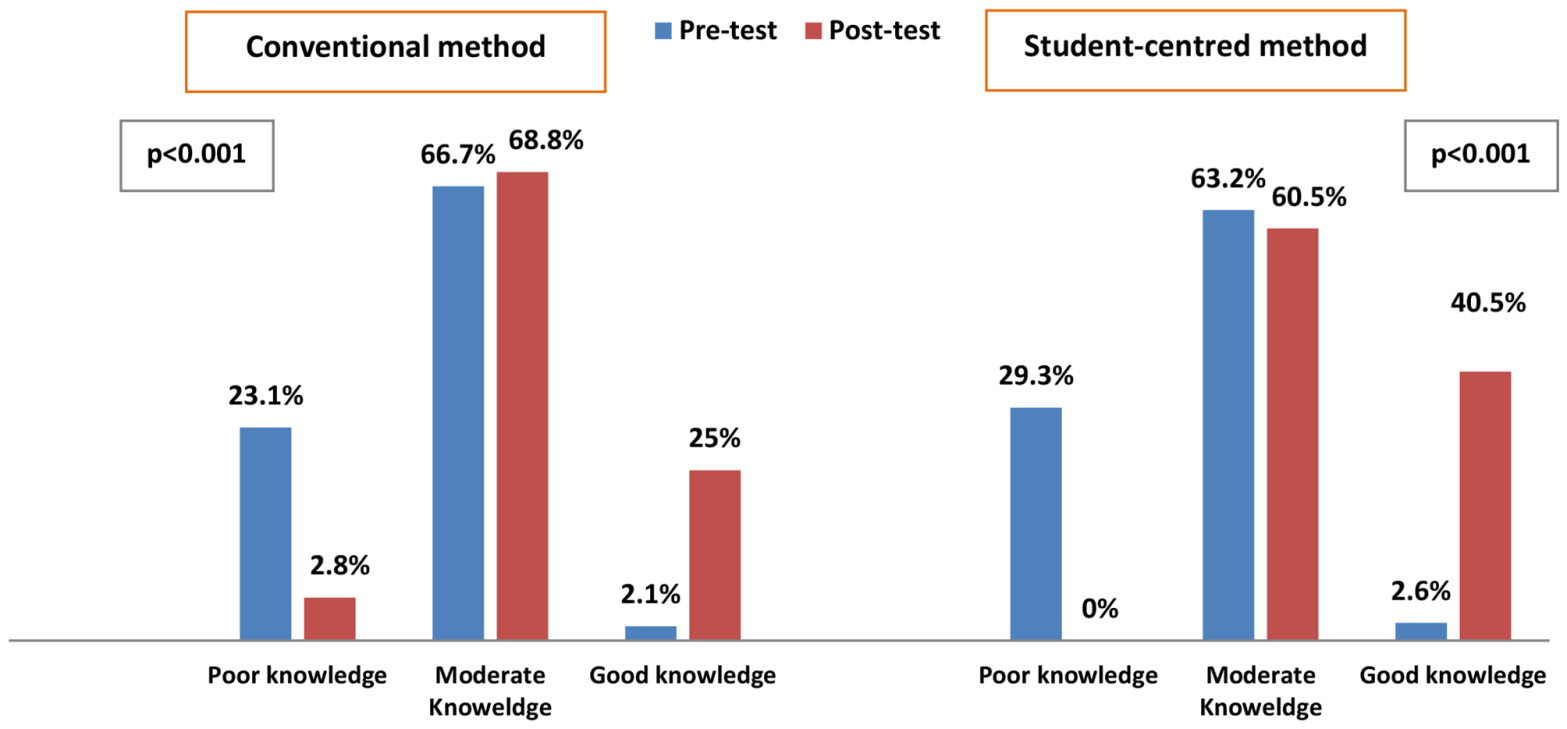
Hand hygiene knowledge levels during different stages of training (N = 203)

As it is described in the Fig. 1, poor knowledge levels decreased from 23.1 to 2.8% (p < 0.001) after conventional training and from 29.3 to 0% (p < 0.001) after SCL method.

The overall improvement in good HH knowledge rates was greater after the SCL method compared to conventional training (40.5% vs. 25%) (Fig. 1).

### Biomedical Waste Management knowledge among students before and after the two training methods

Prior training about BMWM was reported by 11.4% of fifth-year students. Most of participants (94.6%) recognized the biohazard symbol dedicated for infectious waste materials. The cost of infectious waste treatment was underestimated by 51% of medical students.

Table 2 summarizes the influence of both training methods on BMWM scores during different phases of workshops.

In fact, a significant improvement in knowledge levels between pre-test and post-test for the two methods was found. Concerning infectious waste, mean scores were higher after SCL compared to the conventional training in all hazardous waste management steps (total mean scores: 25 ± 3.3 vs. 23.6 ± 5.5, respectively) (Table 2).

Regarding general waste, there was no difference in mean knowledge scores on post training test between conventional and SCL (13 ± 3.4 and 13 ± 2.5, respectively) (Table 2).

The majority of students in our sample stated that combined workshops will be useful for them (90.7% for conventional method and 97.4% for SCL).

**Table 2:** Assessment of Biomedical Waste Management (BMWM) knowledge scores among students at pre- and post-training

Training method		Conventional training (n = 105)			Student-centred training (n = 98)		
Type of biomedical waste		Pre-test mean scores (± SD)	Post-test mean scores (± SD)	P-value	Pre-test mean scores (± SD)	Post-test mean scores (± SD)	P-value
Infectious waste (Maximum score)	Source separation (14)	8.1 ± 3.1	11.8 ± 3.1	< 0.001	8.2 ± 2.6	12.8 ± 1.1	< 0.001
	Destination (14)	8.1 ± 3.2	11.7 ± 2.9	< 0.001	8.9 ± 3.1	12.3 ± 2.6	< 0.001
	Total (28)	16.8 ± 5.3	**23.6 ± 5.5**	**< 0.001**	15.7 ± 4.8	**25 ± 3.3**	**< 0.001**
General waste (Maximum score)	Source separation (8)	5.5 ± 1.8	6.8 ± 1.5	< 0.001	6.5 ± 1.4	6.7 ± 1.1	0.4
	Destination (8)	4.7 ± 2.7	6.1 ± 2.3	0.009	5.3 ± 1.4	6.2 ± 1.1	0.1
	Total (16)	10.2 ± 3.2	**13 ± 3.4**	**< 0.001**	10.1 ± 3.2	**13 ± 2.5**	**0.01**

## Discussion

Our survey is one of the few studies in the Arab world to evaluate knowledge about hand hygiene as well as biomedical waste management among medical students after SCL and comparing its efficiency with the conventional training. Analysis in our study revealed that BMWM and HH score differences between pre-test and post test were statistically significant for both training methods. Nevertheless, the highest mean scores were obtained after SCL.

We found that only 21.7% of participants had undergone prior training in the last three years for HH. Likewise, 36.7% of medical students estimated that they were sufficiently educated about HH according to a Tunisian paper [18]. This proved the necessity of focusing more efforts on providing HH training and rising awareness building in Tunisia. Before training, almost 30% of participants in our sample had poor HH knowledge with no gender difference. Our results are consistent with those reported in numerous studies conducted among medical students in Sri lanka [4] and Pakistan [19]. In fact, at least 31.6% of them had poor HH score for both genders.

In our study, HH knowledge increased from 11.5 ± 2.2 to 13.9 ± 2.3 after conventional training. Based on the same measurement tool and training method, HH scores increased from 8.1 ± 2.9 to 14.6 ± 1.1 among Indian resident doctors [1]. Another interventional study based on lectures, practical demonstrations and HH workshops for medical students in India [12], showed an increase regarding knowledge and practices on post-training evaluation.

Post-test scores were comparatively better for students who had student-centred HH training (14.8 ± 1.9 vs. 13.9 ± 2.3). According to a recent systematic review [9], it is hard to identify the most effective HH teaching method for medical students. The methodological heterogeneity of the included publications and the small number of experimental research conducted mainly in high income countries were the most reported reasons. Providing role modeling trainings with open discussions was the most favourable intervention reported by Pakistani medical students [19]. HH scenario-based simulations for health professionals were effective in reducing the incidence of catheter-related bloodstream infections in Japan [20]. To conclude, coupling interactive teaching sessions and frequent reminders is essential to enhance HH adherence [4].

The majority of students in our sample stated that combined workshops will be useful for them (90.7% for conventional method and 97.4% for SCL). Apart from that, most of them expressed that they would have liked to get these trainings during the early years of their medical curriculum. Thus, based on the Kirkpatrick’s model level one (student’s satisfaction) and level two (skills improvement) [21, 22], both training methods were effective in our study. Nevertheless, there is a need for regular follow-ups and update courses in health care settings in order to guarantee the training success according to level three (behavioral change) and level four (outcome) of the Kirkpatrick’s model. Similarly, Miller [23] claimed that combining the assessment of knowledge with practices in real clinical settings is crucial. His model, called the Miller pyramid, classifies the development of clinical competencies into four levels: the lowest level is ‘knowledge’, the next level is ‘application of knowledge’ evaluated by scenario based exercises, then ‘clinical skills competency’ and ‘clinical performance’, judged by direct observation in hospitals [23].

Based in our results, only 11.4% of students had a prior BMWM training. Likewise, only 4.7% of medical students reported prior training for BMWM in India [1]. Higher rates (44%) were reported by health care professionals in a recent survey performed at two clinical departments in Tunisia [8]. This rate difference is due to regular BMWM audits for practice promotion at University hospitals which are mandatory according to the Tunisian decree of application n ◦ 2008–2745 [8]. Based on a 2019-research in India [24], interns and residents showed a better practice of BMWM compared to students. This can be explained by the lack of training sessions for medical students but also their limited involvement in direct health care provision. In order to fill this gap, combined HH and BMWM workshops have been performed for all students of the fifth year in the Faculty of Medicine of Monastir since 2019. Indeed, there is a considerable role of medical undergraduates in reducing harmful consequences from BMW mishandling [24].

However, it is important to note that despite the Tunisian decree of application n ◦ 2008–2745 mandating training for all health providers, levels of BMWM knowledge and practices remain low among health workers [8]. As a matter of fact, continuous training and supportive supervision for medical students as well as health practitioners should be more encouraged in Tunisia. This will indirectly result in long-term cost-effectiveness [1].

We found an enhancement in BMWM knowledge levels after conventional training (10.2 ± 3.2 vs. 13 ± 3.4; p < 0.001 for general BMW). Our results are in line with those reported in similar quasi-experimental studies among health workers in Tunisia [25] and Nigeria [26] showing that knowledge as well as practical scores increased significantly on post training test. A significant improvement in BMWM levels among resident doctors was reported after structured teaching sessions in India (8 ± 2.2 vs. 9.8 ± 0.5) [1]. After the educational program, the majority of professionals were aware of color-coded bins and safety boxes. These findings reflect the skill enhancement obtained after training sessions.

Regarding infectious BMWM, the overall mean scores were higher after SCL (25 ± 3.3) compared to the conventional method (23.6 ± 5.5). This finding highlights the fact that the SCL was more effective in the achievement of the specific training objectives. In fact, active peer-to-peer presentations and discussions enhance student’s self-reflect, engagement and satisfaction by developing the skills of empathy and collaboration [15]. This can be connected to the absence of perceived position of authority and knowledge disparity between schoolmates.

Unfortunately, it was difficult to compare our findings with those reported in literature. This was related to the limited research mainly evaluating BMWM skills among health professionals. Also, contrarily to HH questionnaires, BMWM measurement tools used in several studies were extremely different. This fact was highlighted in a-2020 systematic review [27] emphasizing the need of developing a validated BMWM instrument.

The current study is subject to some limitations. Indeed, we assessed student’s knowledge and skills via a self-administered questionnaire which might not necessarily reflect an improvement in real-life practice. Thus, a follow-up with regular audits are needed in order to sustain a long-term compliance. Second, the small number of participants may have resulted in limited precision of our findings. In fact, it is difficult to perform a national survey due to the considerable variability in medical curriculum content and education approach between the four faculties of Medicine in Tunisia. Third, it was hard to compare our results because of the lack of studies focusing on BMWM and HH practices among health professionals. To the best of our knowledge, this is the first quasi-experimental survey in North Africa and the Middle East region aiming at training medical students on both HH and BMWM simultaneously and comparing two training methods.

Therefore, such combined trainings may promise a better management of hospital acquired infections and facilitate the infectious pandemic control in the future [1]. According to the International Environmental Technology Centre and the Institute for Global Environmental Strategies [2], capacity building and awareness raising on safe working environment must be mandatory for medical students and healthcare staff but also for the public especially during the current COVID-19 pandemic. Added to that, it is required to continuously evaluate educational trainings in order to determine their value for future health care providers [28].

## Conclusion

This study is one of the first surveys in Tunisia to evaluate the effectiveness of the SCL method on BMWM and HH knowledge compared to the conventional training. In fact, the current study highlighted that the student-centred method resulted in a better improvement in medical students’ knowledge and practices regarding HH as well as infectious BMWM.

These findings make it clear that active training courses for medical students combined to a continuous supervision of practices in health care settings are mandatory to enhance HH and BMWM performance level. In addition, it is necessary to ensure the availability of waste management and HH equipments.

## Data Availability

All data generated or analysed during this study are included in this published article.

## List of abbreviations

HH: Hand Hygiene
BMWM: Biomedical Waste Management
WHO: World Health Organization
SCL: Student’s Centred Learning
